# Reserve size and anthropogenic disturbance affect the density of an African leopard (*Panthera pardus*) meta-population

**DOI:** 10.1371/journal.pone.0209541

**Published:** 2019-06-12

**Authors:** Rasmus Worsøe Havmøller, Simone Tenan, Nikolaj Scharff, Francesco Rovero

**Affiliations:** 1 Center for Macroecology, Evolution and Climate, Natural History Museum of Denmark, University of Copenhagen, Copenhagen, Denmark; 2 Section for Evolutionary Genomics, Natural History Museum of Denmark, University of Copenhagen, Copenhagen, Denmark; 3 Department of Anthropology, University of California, Davis, Davis, United States of America; 4 Vertebrate Zoology Section, MUSE-Museo delle Scienze, Trento, Italy; 5 Tropical Biodiversity Section, MUSE-Museo delle Scienze, Trento, Italy; 6 Department of Biology, University of Florence, Sesto Fiorentino, Italy; CSIRO Townsville Australian Tropical Sciences and Innovation Precinct, AUSTRALIA

## Abstract

Determining correlates of density for large carnivores is important to understand their ecological requirements and develop conservation strategies. Of several earlier density studies conducted globally, relatively few addressed a scale (usually >1000 km^2^) that allows inference on correlates of density over heterogeneous landscapes. We deployed 164 camera trap stations covering ~2500 km^2^ across five areas characterized by broadly different vegetation cover in the Udzungwa Mountains, Tanzania, to investigate correlates of density for a widespread and adaptable carnivore, the leopard (*Panthera pardus*). We modelled data in a spatially explicit capture-recapture framework, with both biotic and abiotic covariates hypothesised to influence density. We found that leopard density increased with distance to protected area boundary (mean±SE estimated effect = 0.44±0.20), a proxy for both protected area extent and distance from surrounding human settlements. We estimated mean density at 4.22 leopards/100 km^2^ (85% CI = 3.33‒5.35/100 km^2^), with no variation across habitat types. Results indicate that protected area extent and anthropogenic disturbance limit leopard populations whereas no support was found for prey availability and trap array as drivers of leopard density. Such vulnerability is relevant to the conservation of the leopard, which is generally considered more resilient to human disturbance than other large cats. Our findings support the notion that protected areas are important to preserve viable population of leopards, increasingly so in times of unprecedented habitat fragmentation. Protection of buffer zones smoothing the abrupt impact of human activities at reserve edges also appears of critical conservation relevance.

## Introduction

Carnivores, and large cats in particular, are not only among the most important flagship species, but also carry out critical ecosystem functions such as herbivore population regulation, which in turn influence ecosystem health [1–3]. Yet, large cats are declining worldwide due to anthropogenic activities, that causes prey decline and habitat loss, as well as pressure from unsustainable trophy hunting and direct persecution [4, 5]. Obtaining accurate density estimates for large carnivores, and understanding the underlying factors, represents a challenging goal in animal ecology [6]. However, this can be difficult because the low abundance and elusive nature of large cats make them inherently difficult to study [2, 7].

Among the large cats, the leopard (*Panthera pardus*) has the widest distribution in the Old World and, while it is still considered common in some areas, its range has declined by 63–75% [8]. Hunting for leopard fur and retaliatory killings for loss of livestock or human attacks have impacted certain leopard population, yet prey depletion and habitat loss are major causes of their decline [8, 9]. Leopards are highly adaptable with regards to habitat, and have been recorded in the widest range of habitat types of any Old World large cat, from mountains, rainforests and deserts, to agricultural and urban areas; they are generally nocturnal and very secretive in nature [8, 10, 11]. Such broad adaptability in diet and habitat, along with their cryptic nature, make deciphering the relative importance of factors affecting density, such as prey abundance, habitat type, and human disturbance, particularly challenging.

Previous studies suggest that multiple correlates are often associated with leopard density. In a review on carnivore abundance correlates by Carbone, Pettorelli [7], prey abundance was highlighted as the most influential factor. Other studies suggested that prey catchability may also be an important factor in fine-scale habitat selection by leopards, in addition to prey availability [12, 13]. Protected area size is another commonly assumed predictor of large carnivore densities and likelihood of their long-term persistence [14, 15]. A study conducted in South Africa addressed edge and disturbance effects on leopard abundance, and found declining density from the core of a protected area to the surrounding, unprotected landscape [16]. A study from Thailand revealed that leopards avoid areas with high human activity, and proximity to trafficked roads [17]. While site use appears to be affected by humans, leopard densities did not appear to be influenced by direct anthropogenic disturbance due to encroachment into a protected area in Nepal [18], and in South Africa some leopard populations had high densities in non-protected areas [19]. In India leopards have adapted to agriculture-dominated landscapes where they occur in relatively high densities [10].

Telemetry information has been commonly used to study resource selection (e.g. [20]). However, while telemetry typically generates fine scale spatial data for a few individuals, spatially explicit capture-recapture (SECR) sampling using camera traps, generates relatively more coarse scale information on several-to-many individuals [21], allowing testing of explicit hypotheses on correlates of density and space use [22, 23]. Despite the vast potential of this approach, to our knowledge there are only a dozen studies that applied robust SECR analyses to leopard density estimation; in addition, a high proportion of these studies have been performed in southern Africa and within a single habitat type [10, 24–31]. Other studies have addressed differences in density between protected and non-protected areas [19, 32], yet the vast majority of leopard density studies have not embraced the potential of SECR analyses by incorporating drivers of species density and detectability.

Here, we used camera trapping across an area of ~2500 km^2^ to estimate the densities of a leopard meta-population inhabiting a heterogeneous landscape in Tanzania, the Udzungwa Mountains. This area is a mosaic of forest blocks interspersed with drier habitats and surrounded by settled and intensively farmed areas, hence it represents a relevant landscape to study factors affecting leopard density. We used a stratified population model in a spatially-explicit capture-mark-recapture framework [33] to test hypotheses on natural and anthropogenic factors driving leopard density at the landscape level. Specifically, we aimed to determine the effects of habitat type, prey availability, distance to water source, sex and distance to protected area boundary on leopard density.

## Material and methods

### Ethics statement

Data collection consisted of non-invasive, remotely set camera traps and did not involve direct contact or interaction with the animals. This research was conducted under research permit numbers 2013-274-NA-2013-111 and 2014-137-ER-2013-111, issued to RWH by the Tanzania Commission for Science and Technology (COSTECH).

### Study area

The Udzungwa Mountains of south-central Tanzania (centred on 7^◦^46’ S, 36^◦^43’ E; elevation 285–2600 m asl, 16,000 km^2^) are part of the Eastern Arc Mountains, a renowned biodiversity hotspot [34, 35]. The Udzungwas consist of closed forest blocks interspersed with drier habitats [36]. It is surrounded by subsistence farming to the north, west and south, and high intensity sugar cane farming to the east, without natural connectivity to other adjacent protected areas [37]. Within Africa, the area is known for its outstanding levels of mammalian richness and endemism [34, 38].

**Fig 1:**
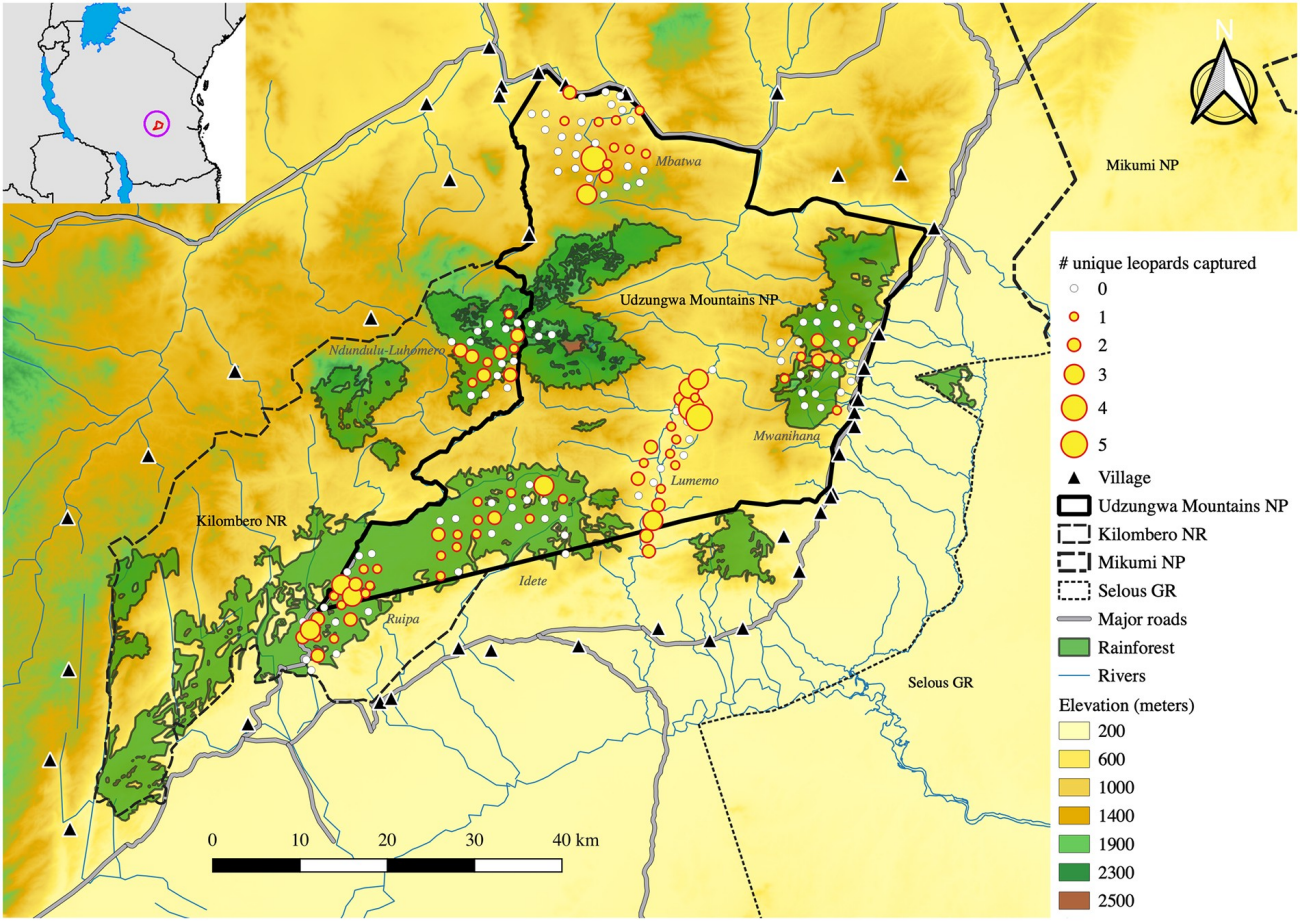
Map of the study area in south-central Tanzania. Six camera trap arrays (*Ruipa*, *Idete*, *Mbatwa*, *Lumemo*, *Ndundulu-Luhomero* and *Mwanihana*) were deployed to detect leopards in five areas with a representative gradient of vegetation cover in the Udzungwa Mountains National Park and Kilombero Nature Reserve. Camera trap sites are indicated by yellow dots, with dot size indicating number of uniquely identified leopards and white dots indicate no detections of leopards. Rainforest areas are indicated in green with a black outline. The elevational gradient ranges from light yellow at low elevation (dry open habitats) to green at higher elevation (moister and mainly forested habitats). The floodplain between Udzungwa Mountains National Park and Selous Game Reserve is an intense agricultural zone with a high human population density.

The northern portion of the Udzungwas is protected by the Udzungwa Mountains National Park (UMNP; 1990 km^2^), where patrolling rangers and remote ranger stations ensure effective ground protection. The UMNP is connected to the south and west by the Kilombero Nature Reserve (1345 km^2^) administered by Tanzania Forest Service, and receives less in situ protection. A strip of intense agriculture, in some places just ~5 km wide, separates the UMNP from the Selous Game Reserve to the east (Fig 1).

As the Udzungwa Mountains consists of a mosaic of habitats with different vegetation cover types [36, 39], we placed camera trap arrays in five areas to sample a representative variation of habitat type (Fig 1; S1 Table): (1) Lowland Afrotropical rainforest in the southern UMNP (300–800 m). (2) Dry grassy *Acacia-Commiphora* woodlands in the northern UMNP, buffered by dry baobab woodlands at low elevation and grasslands at high elevation (500–1900 m). (3) Grassy Miombo woodlands in the central valleys of the UMNP (300–500 m). (4) Ndundulu forest, a block of Afromontane forest west of UMNP in the Kilombero Nature Reserve (1400–2200 m). (5) Mwanihana forest, a rainforest escarpment in the eastern part of UMNP (300–2100 m).

### Camera trapping

We deployed six camera trap arrays covering a total of ~2500 km^2^ in the five areas. Each array consisted of 25–34 pairs of traps (Fig 1) and was surveyed one time each. We sampled in the dry season from August to December 2013 and from June to December 2014. Each station of paired traps operated for an average of 31 days (minimum 12, maximum 49 days; S1 Table). Camera traps were set following a protocol designed for studying leopards in African forests [40]. The average trap spacing was 1.6 km, following a regular spaced grid placed randomly over the study sites, ensuring a sampling grid tight enough to recapture females (with presumed smaller home-ranges than males) in multiple camera traps, while still encompassing an area large enough for a male leopard home-range [40]. Mean edge distance between camera trapping grids was 22.8 km (minimum edge distance 5 km, maximum edge distance 49 km). At each camera trap site, the paired cameras were placed 3–4 m from the centre of an animal trail or track, facing each other at 30–40 cm above ground level. At least one camera-trap per station had a white Xenon flash Cuddeback Ambush (Cuddeback Non Typical Inc., Istanti, WI, USA) and in 87 of 164 stations, the second camera consisted of an infrared camera UOVision 565HD IR+ (UOVision Technology, Shenzhen, China) set on 15-second video recording mode.

Leopards were identified by their unique spot-patterns across their body [41] and presence/absence of external genitalia by two independent observers (RWH and FR). Only individuals deemed adult from their body-size and consistently captured alone were used for subsequent analyses, to avoid non-independence of individual activity centres, e.g. juveniles [22, 23]. For leopards that could only be identified from one flank, gender was used as distinguishing feature from captures of other leopards captured only from the opposite flank of opposite sex.

### Covariates of leopard density

To model leopard density, we derived the following set of covariates across the areas covered by the six trap arrays. We first used Landsat 7 TM and ETM+ satellite imagery to derive at 500 m resolution (1) distance from each camera trap station to the nearest river, (2) distance to the nearest protected area boundary (national park or nature reserve depending on arrays, see Fig 1). Elevation (3) was recorded at each camera trap site using a Garmin GPSMAP 64sc (Olathe, Kansas, USA). Distance to protected area boundary correlated positively (r = 0.65) with distance to the nearest human settlement from each camera trap, thus to avoid collinearity we only used distance to reserve border and considered it a proxy of both anthropogenic disturbance and extent of protected area. The 500 m resolution chosen for the covariates corresponds to the resolution of the state-space adopted in the spatially explicit capture-recapture models (see below), which we defined after testing a range of resolution values that yielded stable parameter estimates and reasonable computational time. We also derived (4) an index of probability of leopard encounter with prey similar to Everatt, Andresen [42] as the array-specific mean estimated occupancy probability of 18 ground dwelling mammals detected by the camera traps [43]. These species were assumed to be potential leopard prey based on dietary studies [44]. In addition, 12 of these species were confirmed to be leopard prey in Udzungwa through DNA analysis of leopard scats ([43]; S2 Table). We estimated mean and array-specific site use probabilities (S3 Table) for the pool of potential prey by fitting a multi-species occupancy model [45] to prey species’ detection/non-detection data. This modelling approach accounts for imperfect detection and solves the ambiguity between species absence and non-detection. We therefore considered occupancy a better state variable for prey abundance than a crude index of captures, as this likely underestimates true abundance due to false negatives [46]. In addition, as we set camera traps to target leopards, detectability of other mammals across sites may vary largely among species, likely resulting in variably biased detection rates; hence we considered it especially critical to use a state variable of prey encounter that is corrected by detectability [47]. We designed our community occupancy model to estimate array-specific mean occupancy values for the pool of prey species, as we assumed that the different habitat types sampled by arrays represent a relevant correlate of variation in the ‘abundance’ of prey species across the study area. However, given that we only had information on prey species at camera trap sites, we could not realistically model prey occupancy across the state-space. Specifically, we modelled the presence/absence *z*_*i*,*j*_ of species *i* at sites *j* as a Bernoulli trial with array-specific (*a*) occupancy probability *ψ*_*i*,*a(j)*_: *z*_*i*,*j*_ *~ Bern*(*ψ*_*i*,*a(j)*_). We constrained the species-specific parameters (i.e., the heterogeneity in occupancy and detection probability) by the assumption of a common normal prior distribution for their logits [48]. For occupancy, we considered an array-specific hyperparameter: logit(*ψ*_*i*,*a(j)*_) = *β*_*i*,*a*_ with *β*_*i*,*a*_ *~ Normal*(*μ*_*ψ*,*a*_, *σ*_*ψ*_*)*, where *μ*_*ψ*,*a*_ is the mean (community) occupancy of prey species in each array, and *σ*_*ψ*_ is the standard deviation. We organized daily detections into a species by sites matrix, with elements *y*_*i*,*j*_, and modelled detections as *y*_*i*,*j*_ *~ Bin*(*k*_*j*_, *p*_*i*,j_*z_*i*,*j*_) where *k*_*j*_ are the sampling occasions per site and *p*_*i*,*j*_ is the detection probability. As we were not interested in modelling array-specific detectability, we modelled detection probability as logit(*p*_*i*,*j*_) = α_i_ with α_i_ *~ Normal*(*μ*_*p*_, *σ*_*p*_), where *μ*_*p*_ is the mean (community) detectability of prey species and *σ*_*p*_ is the standard deviation. We fitted the model using a Bayesian formulation, the Markov chain Monte Carlo, implemented using the program JAGS [49] and executed from R [50]. The model code is provided in S1 Appendix. Finally, given that leopard density associated significantly with the distance to reserve border (see Results), we also controlled whether our prey encounter index may also be associated with this variable, hence potentially confounding the interpretation of effects on leopard density. We therefore ran a second prey occupancy model where the linear predictor for occupancy included an effect of distance to reserve border on array-specific prey site use. Prey site use was found not to be significantly associated with distance to reserve border, therefore we exclude that this effect may also be related to collinear variation in prey site use (see also Discussion).

### Leopard density estimation

We used spatial capture-recapture (SECR) models [22, 23] to account for animal movement in density estimation, regarding array-specific data as samples of independent populations. This assumption is supported by the absence of individuals recorded in more than one trap array. SECR models allow study of the distribution of individuals (i.e. density) while accounting for encounter probability (p) that declines with distance between an individual activity centre (s) and a detector (j). We used a half-normal encounter model where detectability p is a function of the baseline encounter probability (*p*_*0*_) and the spatial scale parameter (σ), which determines how encounter probability decreases with an increase in the distance between trap j and activity centre s_i_.

Both homogeneous and inhomogeneous point process models were fitted to study the distribution of individual activity centres within a defined state-space S, depending on the absence or presence, respectively, of spatially explicit covariates on density. We fitted a stratified population model [22, 23] to data grouped by trap array, where array-specific population size was assumed as N_r_ ~ Poisson(Λ_r_), where Λ_r_ is the expected number of activity centres in the state-space, or region, surrounding array r, with r = 1, …, R = 6. We investigated the effects of covariates (‘COV’, see previous section for details) on leopard density and detectability by testing different hypotheses. First, we defined the best structure of the encounter model by assessing the effect of the following covariates on the baseline encounter probability (*p*_*0*_): (i) trap array, as a proxy of habitat type and seasonality (i.e. temporal variation in sampling), (ii) distance of trap j to the nearest river, (iii) distance to reserve boundary, (iv) camera trap type and (v) sex, for testing the effect of different probabilities of detections of males and females. The same covariates, with the additional array-specific effect of prey encounters, along with sex, which has been found to produces better estimates of density due to differences in home-ranges sizes [51], were used as competing predictors for modelling the scale parameter (σ). The general formulation of the linear predictors for two parameters of the encounter model, for individual i in trap j of array r, was as follows:

$$\text{logit}\left(p_{0,\text{jr}}\right)=\alpha_{0}+\alpha_{\text{COV}}{\text{COV}}_{\text{jr}}$$ (1)

$$\text{log}\left(\sigma_{\text{jr}}\right)=\delta_{0}+\delta_{\text{COV}}{\text{COV}}_{\text{jr}}.$$ (2)

Competing encounter models based on plausible combination of different covariates (‘COV’).

Specifically, we expected encounter rate to decline (i) with increasing distance to rivers, as waterways are frequently used as travelling routes and foraging areas [52, 53], and (ii) with increasing distance to reserve boundary, in relation to possible behavioural effects induced by an increase of anthropogenic disturbance close to the reserve boundary [13, 54]. We also expected leopards to move less (thus having smaller home range size), in dense versus open habitats, as the species has been found to prefer dense habitat for hunting and thus would have to travel smaller distances in search of optimal hunting grounds [13]. In addition, we expected leopard space usage to be (i) positively correlated with distance to the nearest river, as rivers may represent good hunting grounds [53], and (ii) positively correlated with distance from reserve boundary, where anthropogenic disturbance is higher.

We were interested in modelling density as a function of spatially-varying covariates and used a discrete representation of the state space with the centre points of each pixel g(r) (with g(r) = 1, …, G_r_) in the state-space (region) surrounding trap array (r). The expected number of activity centres in the state-space surrounding trap array (r) was modelled in relation to (i) elevation, (ii) distance to the nearest river, (iii) distance to reserve boundary, (iv) prey encounter index (occupancy probability of prey community; S3 Table), (v) trap array, and (vi) sex (S1 Table). Distance to river was intended as a proxy to major traveling routes used by leopards [52, 53]. In addition, it may also indicate proximity to optimal hunting grounds. As elaborated above, we considered distance to reserve boundary a proxy for both reserve size (i.e. remoteness) and human disturbance; as settlements and farms occur right outside protected areas, we assumed human encroachment and other forms of disturbance to be more intense near boundaries [16, 17, 55]. We expected density to be negatively correlated with elevation, as area declines with increased altitude and because high-altitude habitats (1400–2600 m) may hold sub-optimal abundance and low diversity of prey, that may be limiting leopard densities [56]. We hypothesised a negative correlation for density with increasing distance to permanently flowing rivers as an indication of preferred hunting ground and travel routes [52]. We expected the mean of the prey encounter index as proxy for prey abundance to be positively correlated with leopard density, matching evidence for other large carnivores and for leopards [57, 58]. We assumed leopard densities to be higher in low elevation habitats with denser, arboreal vegetation cover (closed lowland forest versus open woodland and wooded grassland) as these may hold more optimal hunting grounds [13]. We assumed different densities between male and female leopards as overlap in home-ranges of adult males is unusual and their home-ranges are normally larger than females [59]. Expected number of activity centres were modelled the in the state-space of array r in relation to the different covariates (‘COV’) as follows:

$$\text{log}\left(\Lambda_{\text{g}(\text{r})}\right)=\beta_{0}+\beta_{\text{COV}}{\text{COV}}_{\text{g}(\text{r})}+\beta_{\text{COV}}{\text{COV}}_{\text{r}}$$ (3)

where covariates can be either spatially explicit (i.e. rasterised, ‘COV_g(r)_’) or region (i.e. survey or array) specific (‘COV_r_’). We first defined the best structure for the encounter model (36 competing models, S4 Table), while considering survey-specific densities, and then tested hypotheses on the correlates of leopard density (13 competing models, S5 Table) while keeping the best encounter structure constant. We set a 6 km buffer around each trap array based on ridged density estimates descending from 30 km, and based inference on maximum likelihood estimates for leopard density using the R package ‘secr’ v. 3.2.0 [33]. Capture histories were based on daily sampling occasions. We calculated the Akaike Information Criterion (AIC) for each candidate model and used the difference among values (ΔAIC) to rank models. According to Burnham and Anderson [60], we examined models within 2 ΔAIC units to assess whether they differed from the best model by one parameter and had similar values of the maximized log-likelihood as the best model. In our case, we did not consider the top two models being supported, despite being within 2 ΔAIC units, because the second-best model had an additional parameter, yet the log-likelihood value was not improved. We also considered 85% confidence intervals for parameter estimates, that are compatible with information-based model selection to evaluate potential uninformative parameters Arnold [61]. We derived density surface estimates from models that scored two points within their estimated ΔAIC [62]. We also derived home range size estimates (in km^2^) based on Royle, Chandler [22], using the formula π (σ √5.99)^2^, where σ is the spatial scale parameter in km, interpreted as the radius of the bivariate normal model of space use. The product (σ √5.99) is the 95% of space used by an individual from the home-range centre. The value √5.99 derives from the fact that the square of the distances between trap location and individual activity centres used in the Gaussian encounter probability model have a chi-square distribution with 2 degrees of freedom which, for α = 0.05, corresponds to √5.99.

## Results

We accumulated a sampling effort of 5038 camera trap days and obtained 185 leopard events.

Overall, 62 individuals were identified from all six surveys (median 10, minimum 5, maximum 15; S1 Table), of these 62 individuals four were identified as juveniles and never captured independently and were therefore excluded from the dataset, bringing the total number of individuals used in the SECR analyses to 58. The 58 adult leopards were captured in 48.6% of the 164 camera trap stations (S1 Table). No discrepancies were found between the two persons reviewing the photos (RWH and FR). Individuals identified from just one flank made up <13% of individuals (8 of 62) and all of these were single records (~4% of the total dataset) were confidently identified as unique, as males were captured on their right flank, and females were captured on their left flank. Five captures were of unidentifiable leopards and were not included in the dataset.

Based on AIC weight, the most parsimonious encounter model included an effect of distance to the nearest river on the baseline encounter probability (*p*_*0*_) and array-specific scale parameter *σ* (Table 1).

**Table 1:** Results of encounter models evaluated to estimate leopard density. Summary of the encounter model selection for the 36 competing models based on different covariate combinations on the baseline encounter probability (*p*0) and scale parameter (*σ*) of leopards detected by camera traps in the Udzungwa Mountains of Tanzania. Parameters were run either as single or dual covariables. Number of parameters, log likelihood, AIC and AIC weight is listed for each model.

Model	*p0*			Σ			No. of parameters	Log. likelihood	AIC	AIC weight
ggs1	Distance to river			Trap array			14	-1023.343	2074.687	0.3423
ggs2	Null			Trap array			13	-1025.376	2076.751	0.2628
ggs3	Distance to river	+	Distance to boundary	Trap array			15	-1021.873	2073.746	0.2333
ggs4	Distance to boundary			Trap array			14	-1024.808	2077.617	0.0791
ggs5	Camera trap type			Trap array			14	-1025.183	2078.365	0.0544
ggs6	Distance to river			Prey encounter index			10	-1033.179	2086.359	0.0136
ggs7	Distance to river	+	Distance to boundary	Prey encounter index			11	-1032.11	2086.219	0.0085
ggs8	Distance to river			Prey encounter index	+	Distance to river	11	-1033.051	2088.102	0.0033
ggs9	Distance to boundary			Prey encounter index			10	-1034.761	2089.522	0.0028
ggs10	Trap array			Trap array	+	Distance to boundary	19	-1018.171	2074.342	0
ggs11	Distance to boundary			Prey encounter index	+	Distance to river	11	-1033.861	2089.723	0
ggs19	Trap array			Trap array			18	-1020.808	2077.615	0
ggs12	Null			Null			8	-1039.493	2094.986	0
ggs13	Distance to river			Distance to river			10	-1036.937	2093.875	0
ggs14	Trap array	+	Distance to boundary	Trap array			19	-1020.094	2078.188	0
ggs15	Trap array	+	Distance to river	Trap array			19	-1020.113	2078.226	0
ggs16	Distance to river	+	Distance to boundary	Distance to river			11	-1035.707	2093.415	0
ggs17	Distance to river	+	Distance to boundary	Distance to boundary			11	-1035.989	2093.977	0
ggs18	Trap array	+	Camera trap type	Trap array			19	-1020.778	2079.557	0
ggs20	Trap array			Trap array	+	Distance to river	19	-1020.795	2079.59	0
ggs21	Distance to boundary			Distance to river			10	-1037.783	2095.565	0
ggs22	Trap array			Prey encounter index			14	-1032	2092	0
ggs23	Distance to boundary			Distance to boundary			10	-1039.064	2098.129	0
ggs24	Trap array			Null			13	-1035.59	2097.181	0
ggs25	Trap array			Distance to river			14	-1034.535	2097.069	0
ggs26	Trap array			Distance to boundary			14	-1034.864	2097.727	0
ggs27	Sex			Trap array			14	-1062.545	2153.09	0
ggs40	Distance to river			Sex	+	Trap array	15	-1061.271	2152.542	0
ggs42	Null			Sex	+	Trap array	14	-1063.362	2154.724	0
ggs43	Sex			Sex	+	Trap array	15	-1062.437	2154.874	0
ggs41	Distance to boundary			Sex	+	Trap array	15	-1062.769	2155.539	0
ggs44	Camera trap type			Sex	+	Trap array	15	-1063.185	2156.37	0
ggs39	Distance to river	+	Distance to boundary	Prey encounter index	+	Sex	12	-1070.494	2164.989	0
ggs32	Trap array			Trap array	+	Sex	19	-1057.454	2152.909	0
ggs28	Distance to river			Sex			10	-1075.787	2171.573	0
ggs33	Distance to river	+	Distance to boundary	Sex			11	-1074.816	2171.632	0
ggs31	Null			Sex			9	-1078.037	2174.074	0
ggs37	Distance to river	+	Distance to boundary	Distance to river	+	Sex	12	-1074.396	2172.791	0
ggs29	Distance to boundary			Sex			10	-1077.787	2175.575	0
ggs38	Distance to river	+	Distance to boundary	Distance to boundary	+	Sex	12	-1074.815	2173.629	0
ggs34	Camera trap type			Sex			10	-1078.018	2176.037	0
ggs35	Trap array	+	Distance to river	Sex			15	-1072.338	2174.676	0
ggs30	Trap array			Sex			14	-1074.557	2177.114	0
ggs36	Trap array	+	Camera trap type	Sex			15	-1074.546	2179.091	0

**Fig 2:**
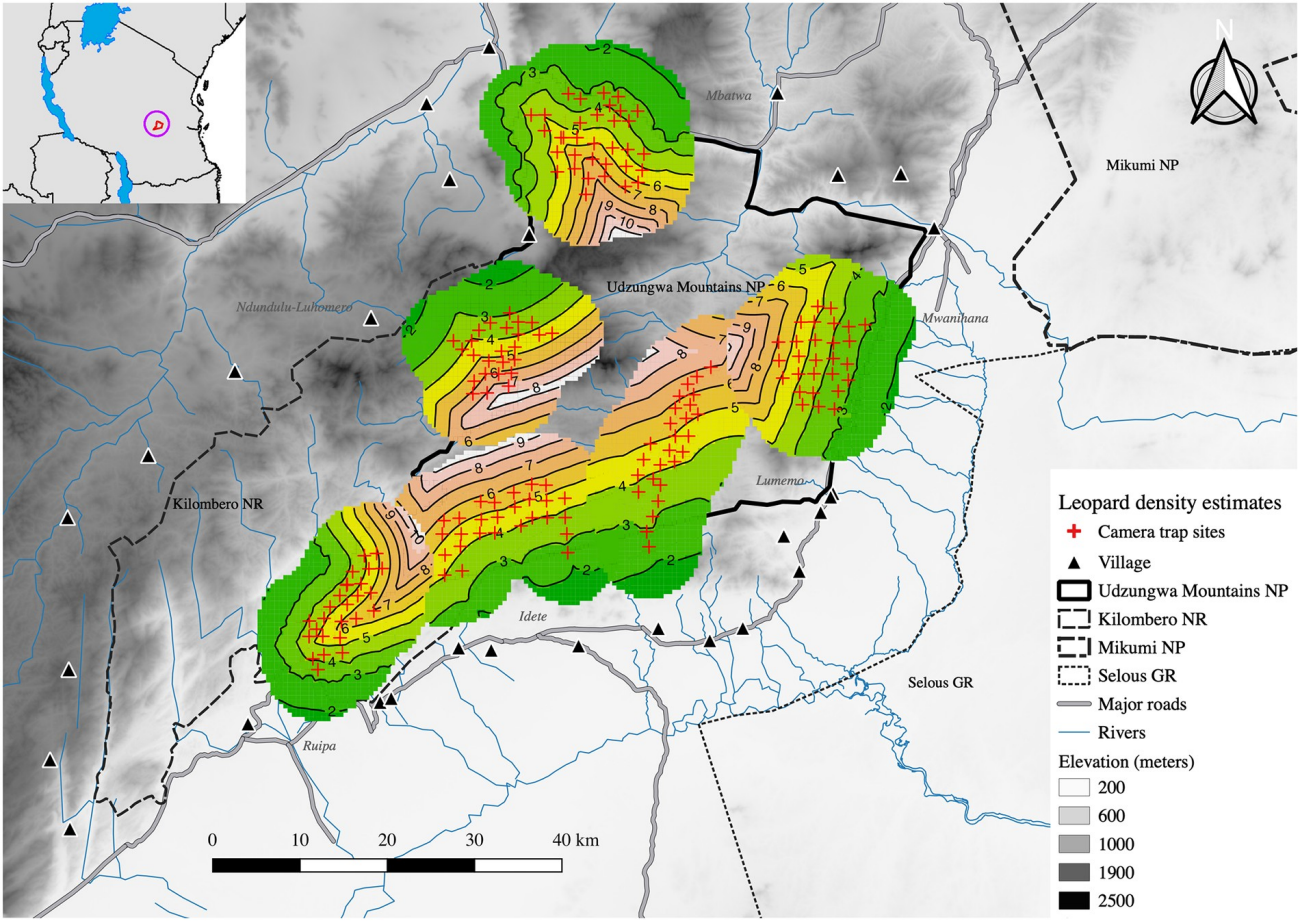
Spatially explicit leopard density map. Expected leopard density (individuals/100 km^2^) in the Udzungwa Mountains, Tanzania, as the predicted density surface for the state-space S superimposed over each trap array. Densities are scaled individually for each trap array with green colour indicating low densities, increasing to higher densities with warmer reddish colours. Camera trap sites are indicated as red crosses.

A single model that included distance to reserve boundary (model Md7 in Table 2) was most supported based on AIC score. Another model (Md15 in Table 2), with an additional effect of prey encounters, was ranked within 2 AIC units (ΔAIC = 0.771) of the model with lowest AIC (model Md7). However, since Md15 differs from the best model by one parameter and have essentially the same value of the maximized log-likelihood (-1025.334 vs -1024.719), indicating the fit is not improved, we considered Md15 not adequately supported. Furthermore, the 85% CI for the estimate of the effect of prey encounters on leopard density in model Md15 encompassed zero (0.093 mean; 85%CI = -0.027‒0.214) supporting the uninformative role of this covariate. We thus based our inference on the most supported model (Md7 in Table 2), which suggested that leopard density was positively influenced by distance to reserve boundary, with the related parameter β_d2boundary_ equal to 0.44, (0.15‒0.73, mean and 85% CI) (Table 3, Fig 2).

**Table 2:** Results of density estimation models. Ranking of the 13 models evaluated for density estimation of leopards detected by camera traps in the Udzungwa Mountains of Tanzania. The highest scoring model (Md7) included the covariates of distance to boundary on Density, distance to river on encounter probability (*p0*) and trap array on the scale parameter (*σ*). Number of parameters, log. likelihood, AIC score, ΔAIC and AIC weight is listed for each model.

Model	Density			*p*0	σ	No. parameters	Log. likelihood	AIC	AIC weight
Md7	Distance to boundary			Distance to river	Trap array	10	-1025.334	2070.668	0.423
Md15	Prey encounter index	+	Distance to boundary	Distance to river	Trap array	11	-1024.719	2071.439	0.167
Md2	Null			Distance to river	Trap array	9	-1027.797	2073.594	0.158
Md9	Elevation			Distance to river	Trap array	10	-1027.029	2074.057	0.078
Md23	Prey encounter index			Distance to river	Trap array	10	-1027.25	2074.5	0.062
Md8	Distance to river			Distance to river	Trap array	10	-1027.446	2074.891	0.051
Md13	Prey encounter index	+	Elevation	Distance to river	Trap array	11	-1026.575	2075.151	0.026
Md14	Prey encounter index	+	Distance to river	Distance to river	Trap array	11	-1026.66	2075.319	0.024
Md4	Trap array	+	Distance to boundary	Distance to river	Trap array	15	-1020.902	2071.805	0.008
Md1	Trap array			Distance to river	Trap array	14	-1023.343	2074.687	0.004
Md5	Trap array	+	Distance to river	Distance to river	Trap array	15	-1022.447	2074.893	0
Md6	Trap array	+	Elevation	Distance to river	Trap array	15	-1022.803	2075.605	0
Md3	Sex			Distance to river	Trap array	10	-1066.593	2153.185	0

**Table 3:** Results of the leopard density model. Maximum likelihood estimates for leopards detected by camera trapping in the Udzungwa Mountains of Tanzania. Estimates were derived from the most supported model (Md7 in Table 3) which included covariates on distance to boundary for the density parameter, distance to river on the baseline encounter probability (*p0*) and trap array on the scale parameter (*σ*).

Parameter	Description (scale)	Mean	SE	Lower 85% CI	Upper 85% CI
*β*_0_	Intercept of density (log)	-7.770	0.164	-8.007	-7.534
*β*_d2boundary_	Effect of distance to reserve boundary on density (log)	0.439	0.203	0.147	0.731
*α*_0_	Intercept of baseline encounter probability *p*_0_ (logit)	-3.392	0.135	-3.586	-3.198
*α*_d2river_	Effect of distance to river on the baseline encounter probability *p*_0_ (logit)	-0.211	0.106	-0.364	-0.059
*δ*_0_	Intercept of scale parameter *σ* of the encounter model (log)	7.517	0.144	7.310	7.725
*δ*_Lumemo_	Survey effect on the scale parameter *σ* (log)	0.283	0.180	0.023	0.542
*δ*_Mbatwa_	Survey effect on the scale parameter *σ* (log)	0.033	0.167	-0.208	0.273
*δ*_Mwanihana_	Survey effect on the scale parameter *σ* (log)	-0.471	0.198	-0.756	-0.186
*δ*_Ndundulu_	Survey effect on the scale parameter *σ* (log)	-0.348	0.171	-0.595	-0.102
*δ*_Ruipa_	Survey effect on the scale parameter *σ* (log)	0.206	0.177	-0.049	0.462

This effect translated into predicted densities that varied from approximately 2/100 km^2^ along the reserve border to over 8/100 km^2^ in the reserve interior (Fig 2). Mean density for the total area surveyed was estimated to be 4.22/100 km^2^ (85% CI = 3.33‒5.35/100 km^2^); mean density in this case is the exponential of the intercept of density (*β*_0_ in equation [3]), which corresponds to density evaluated at the mean value of the covariates that were standardized to have mean 0 and unit variance.

Intercept of the baseline encounter probability, on probability scale, was 0.030 (0.022‒0.040). In addition, results suggest a negative relationship between encounter probability and distance of camera trap to the nearest river (α_d2river_ = -0.21, -0.36‒ -0.01, Table 3). Trap array-specific estimates of the spatial scale parameter of the half-normal encounter model (S6 Table) were used to derive array-specific estimates of 95% home range sizes, which varied from a minimum of 25 km^2^ in Mwanihana to a maximum of 112 km^2^ in Lumemo (mean 66 km^2^) (S7 Table).

## Discussion

### Correlates of leopard density at the landscape level

We analysed factors affecting the spatial variation of leopard density within a heterogeneous landscape and found that leopard density was significantly associated with distance to reserve boundary, which we considered a proxy for the extent of protected area and decreasing human disturbance [63]. The Udzungwa Mountains National Park and adjacent Kilombero Nature Reserve form a relatively large protected area (3335 km^2^). Concomitantly, evidence suggests that in proximity of reserve boundaries human disturbance increases, in the form of firewood collection, selective pole and timber logging, poaching and charcoal production [43, 64]. Importantly, by assessing that prey occupancy model was not associated with distance to reserve boundary (see Methods) we could exclude that increasing leopard density away from reserve borders is mediated by an effect of increasing prey encounter probability.

Our findings partially mirror those from a study in South Africa, where edge effects and higher mortality rates were associated with lowered densities of leopards outside the protected area relative to inside [16]. Our results are similar to those of a study in Thailand, in which leopards were reported to avoid roads and areas with high human activity compared to undisturbed areas and became more diurnal when human presence became limited [54]. In a broader perspective, the magnitude of the effect of distance to reserve boundary fits the known requirement of large carnivores, for large areas of protected habitat [65]. We elaborate in Methods the value of this metric of occurrence that accounts for imperfect detection, a consistent issue when sampling elusive mammals in dense habitats, e.g. Dorazio, Royle [45], and therefore standardizes occupancy estimation across arrays that differ markedly in habitat type and across species. We acknowledge that a limitation of this metric is that it does not measure actual prey abundance, biomass or leopard prey preference, and it may also under-represent the full spectrum of prey species. Our results partially mirror those of Balme, Hunter [13], that did not find prey abundance to be the most important factor for a population of leopards in South African savannah woodland.

We deployed six camera trap arrays covering five areas representative of the variation in vegetation cover, from montane to lowland rainforest, dry forest and wooded grassland (Fig 1; S1 Table). However, we did not find support for a substantial variation of leopard density across these arrays. This result supports the notion of leopards being habitat generalists [59], and in our case study their apparent flexibility in respect to vegetation cover and the fact that leopards are the most abundant large carnivore in Udzungwa (spotted hyenas [*Crocuta crocuta*] occur in lower density and lions [*Panthera leo*] are only occasionally recorded [43]), thus leopards are not constrained by interactions with other large carnivores.

We found that baseline encounter probability (*p0*) was positively correlated with proximity to rivers, while space-usage (σ) varied with trap array. Higher encounter probability close to waterways may be related to habitat structure, with large and frequently used trails cutting across dense vegetation may result in optimal detection of animals by camera traps, as opposed to less dense habitats. Travelling along rivers is also known to be more energy efficient and favoured places for scent marking and hunting [40, 52, 53]. Higher density of leopards close to rivers may increase encounter probability if the two variables are positively correlated. However, we did not find support for a significant relationship between density and distance to rivers, potentially because trails along rivers are used as travel routes and boundaries for many individuals.

### Conservation implications

We considered a suite of natural and anthropogenic factors hypothesised to affect leopard densities in a complex landscape with different habitat types and found that distance to protected area boundary was the single most influential factor affecting leopard density. We also found that the importance of this factor, overwhelmed the influence of prey encounters and of areas with different vegetation cover, as neither had an effect on leopard densities. These results support the notion of high flexibility of the leopard [44]. However, we note that no individual leopards were recaptured between the sampling grids despite their relative proximity. Our mean population density estimate of 4.22 leopards/100 km^2^ appears in the mid-range when compared to densities from other areas of Africa, where high density estimates of 12.03/100 km^2^ are known from Kenya [31] and low estimates of 0.59/100 km^2^ are known from Namibia [27]. In Udzungwa, leopards are reported as extremely rare or locally extinct in the least protected parts of the Kilombero Nature Reserve [66] and in smaller and poorly protected forests in the range, such as Uzungwa Scarp [67]. Indeed recent research shows that the reserves adjacent to UMNP have much lower mammalian abundance and species richness and that this is associated with their level of protection [64]. Despite of the intense patrolling and permanently manned ranger stations in the UMNP, we captured photographs of armed poachers across all arrays, and recorded camps, snares, carcasses (predominantly elephants, but also other animals including leopard) across the UMNP.

Leopards disappearance in parts of the Udzungwas has been attributed to direct hunting and prey depredation [68], mirroring findings from the Congo [69] and South Africa [16]. While our study shows populations in the highest category of protected areas are in the mid-range densities reported in Africa, the regional metapopulation could be at risk if they lose connectivity with the major adjacent ecosystems of Selous and Ruaha. While Selous Game Reserve is in close proximity (min. 6.4 km, Fig 1), the intersecting area is intensively farmed and settled, hence leopard movements between these protected areas may be absent or only sporadic. Our findings carry important conservation implications, which are related to the need for maintaining large areas of continuous, well-protected habitat to preserve viable populations of large carnivores [14]. This becomes even more imperative given the ever increasing habitat fragmentation that terrestrial mammals face globally [70]. Wildlife Management Areas (WMAs), i.e., areas co-managed with local communities [71], have been proven effective in protecting other wildlife species in Tanzania [72]. This could potentially be a useful management strategy in the Udzungwas for establishing buffer zones in the community land surrounding the nature and forest reserves. This would ensure greater protection along the currently abrupt edges between reserves and human settlements [73]. Given that similarly to all other large cats leopards are suffering from population decline and range loss [8], it is imperative that their strongholds are efficiently protected and their populations monitored to ensure their long-term survival.

## Supporting information

S1 Appendix Model code. (DOCX)

S1 Table Summary of sampling efforts and areas. Details of camera trap surveys in the Udzungwa mountains of Tanzania per each camera trap array: dominant habitat type, number of paired camera trap stations, camera trap days and survey period. Camera-trap stations were retrieved after ~30 days and each survey had a mean of 27 camera traps (range 25–34).
(DOCX)

S2 Table Potential leopard prey species. List of prey species detected by camera trapping in the Udzungwa Mountains of Tanzania during the leopard survey and assumed to be potential prey. The list includes daily detections that were used to estimate mean occupancy probability for each of the six trap array (see S1 Table).
(DOCX)

S3 Table Summary results of the community occupancy model. Posterior Bayesian distributions and quantiles of mean occupancy (*ψ*) and detectability (*p*) for the community of potential leopard prey species detected by camera trapping in the Udzungwa mountains of Tanzania (see text for details).
(DOCX)

S4 Table List of competing encounter models. List of competing encounter models with the specific hypotheses tested on the baseline encounter probability (*p*_*0*_) and the scale parameter (*σ*).
(DOCX)

S5 Table List of competing density models. List of competing models for leopard density, as the combination of different covariates in the density linear predictor.
(DOCX)

S6 Table Survey-specific estimates of the scale parameter (*σ*) of the half-normal encounter model. Estimates are expressed in meters (m) with upper and lower confidence intervals (CI).
(DOCX)

S7 Table Summary of leopard home range estimates. 95% home-range estimates in km^2^ for leopards in the Udzungwa mountains of Tanzania in each trap array based on spatially explicit capture-recapture models.
(DOCX)
