# Supplementary material for: Reserve size and anthropogenic disturbance affect the density of an African leopard (Panthera pardus) meta-population
Source: PLoS One. 2019 Jun 12;14(6):e0209541. doi: 10.1371/journal.pone.0209541 (PMC6561539; doi:10.1371/journal.pone.0209541)
Supplement: S1 Appendix — (DOCX) [file pone.0209541.s001.docx]

**S1 Appendix. Model code.** R and JAGS script for the multi-species community occupancy model used to estimate array-specific prey occupancy values.

# load data

load("prey species model data.RData")

# data are a species by site matrix of detections and a vector of camera days per site, with the structure shown hereafter:

str(Y)

int [1:18, 1:164] 9 0 0 0 0 0 0 0 0 2 ...

- attr(*, "dimnames")=List of 2

..$ : NULL

..$ : chr [1:164] "X1" "X2" "X3" "X4" ...

str(effort)

int [1:164] 30 30 30 30 30 30 29 29 30 29 ...

# load array specification (array-specific site index)

session_vect <- c(1,1,1,1,1,1,1,1,1,1,1,1,1,1,1,1,1,1,1,1,1,1,1,1,1,1,

2,2,2,2,2,2,2,2,2,2,2,2,2,2,2,2,2,2,2,2,2,2,2,2,2,

3,3,3,3,3,3,3,3,3,3,3,3,3,3,3,3,3,3,3,3,3,3,3,3,3,3,3,3,3,3,3,3,3,3,

4,4,4,4,4,4,4,4,4,4,4,4,4,4,4,4,4,4,4,4,4,4,4,4,4,4,

5,5,5,5,5,5,5,5,5,5,5,5,5,5,5,5,5,5,5,5,5,5,5,5,5,

6,6,6,6,6,6,6,6,6,6,6,6,6,6,6,6,6,6,6,6,6,6,6,6,6,6,6,6)

# load libraries

library(snow)

library(rjags)

library(dclone)

# set seed

set.seed(1980)

# BUGS model

modelFilename = "smsom_no_DA.txt"

# load libraries

library(snow)

library(rjags)

library(dclone)

model {

# Priors for community-level parameters

for (a in 1:n.arrays){

psi.mean[a] ~ dunif(0,1) # array-specific occupancy (logit scale)

beta[a] <- log(psi.mean[a]) - log(1-psi.mean[a]) # logit(psi.mean[a])

}

p.mean ~ dunif(0,1) # mean community detectability (logit scale)

alpha <- log(p.mean) - log(1-p.mean) # logit(p.mean)

sigma.psi ~ dunif(0,10)

sigma.p ~ dunif(0,10)

tau.psi <- pow(sigma.psi,-2)

tau.p <- pow(sigma.p,-2)

# Likelihood

for (i in 1:N) {

for (a in 1:n.arrays){

# occupancy process

phi[i,a] ~ dnorm(beta[a], tau.psi)

logit(psi[i,a]) <- phi[i,a]

}

# detectability

eta[i] ~ dnorm(alpha, tau.p)

logit(p[i]) <- eta[i]

# state process

for (j in 1:n.site) {

Z[i,j] ~ dbern(psi[i,arr[j]])

mu.p[i,j] <- p[i]*Z[i,j]

Y[i,j] ~ dbin(mu.p[i,j], K[j])

}

}

}

# number of sampling occasions for each trap

K <- effort$ndays

# number of traps

nsites <- dim(Y)[2]

# number of observed species

nspecies <- dim(Y)[1]

# number of arrays

n.arrays <- length(unique((session_vect)))

# Parameters monitored

parameters <- c("psi.mean","sigma.psi","p.mean","sigma.p")

# data

bugs.data <- list(N=nspecies,n.site=nsites,K=K,Y=Y, Z=(Y>0)*1,n.arrays=n.arrays,arr=session_vect)

# initial values

inits <- function() { list(psi.mean=runif(n.arrays), p.mean=runif(1),

sigma.psi=runif(1,0,4), sigma.p=runif(1,0,4))}

#mcmc settings

n.adapt <- 5000 #pre-burnin

n.update <- 10000 #burnin

n.iter <- 30000 #iterations post-burnin

thin <- 10

chains<-3

# run the model and record the run time

cl <- makeCluster(chains, type = "SOCK")

start.time = Sys.time()

out <- jags.parfit(cl, data = bugs.data,

params = parameters,

model = "smsom_no_DA.txt",

inits = inits,

n.adapt = n.adapt,

n.update = n.update,

n.iter = n.iter,

thin = thin, n.chains = chains)

end.time = Sys.time()

elapsed.time = difftime(end.time, start.time, units='mins')

cat(paste(paste('Posterior computed in ', elapsed.time, sep=''), ' minutes\n', sep=''))

stopCluster(cl)

# End of script
