# Supplementary material for: Reserve size and anthropogenic disturbance affect the density of an African leopard (Panthera pardus) meta-population
Source: PLoS One. 2019 Jun 12;14(6):e0209541. doi: 10.1371/journal.pone.0209541 (PMC6561539; doi:10.1371/journal.pone.0209541)
Supplement: S1 Table — Details of camera trap surveys in the Udzungwa mountains of Tanzania per each camera trap array: dominant habitat type, number of paired camera trap stations, camera trap days and survey period. Camera-trap stations were retrieved after ~30 days and each survey had a mean of 27 camera traps (range 25–34). (DOCX) [file pone.0209541.s002.docx]

| **Trap array** | **Dominant habitat type** | **No. stations** | **Survey period** | **No. events** | **Camera traps days** | **No. individuals** | **No. individuals captured once** |
| --- | --- | --- | --- | --- | --- | --- | --- |
| Ruipa | Lowland rainforest | 26 | Sep. - Oct. 2013 | 41 | 775 | 12 | 4 (33%) |
| Idete | Lowland rainforest | 27 | Oct. - Dec. 2013 | 22 | 744 | 9 | 3 (33%) |
| Mbatwa | *Acacia-Commiphora* | 34 | Jun. - Jul. 2014 | 22 | 985 | 6 | 2 (33%) |
| Lumemo | Miombo woodland | 26 | Jul. - Sep. 2014 | 53 | 887 | 15 | 4 (26%) |
| Ndundulu-Luhomero | Montane rainforest | 25 | Sep. - Oct. 2014 | 33 | 774 | 11 | 4 (36%) |
| Mwanihana | Rainforest escapement | 28 | Oct. - Dec. 2014 | 14 | 873 | 5 | 1 (20%) |
|  | Total | 164 |  | 185 | 5038 | 58 | 18 (31%) |
