# Supplementary material for: Reserve size and anthropogenic disturbance affect the density of an African leopard (Panthera pardus) meta-population
Source: PLoS One. 2019 Jun 12;14(6):e0209541. doi: 10.1371/journal.pone.0209541 (PMC6561539; doi:10.1371/journal.pone.0209541)
Supplement: S2 Table — List of prey species detected by camera trapping in the Udzungwa Mountains of Tanzania during the leopard survey and assumed to be potential prey. The list includes daily detections that were used to estimate mean occupancy probability for each of the six trap array (see S1 Table). (DOCX) [file pone.0209541.s003.docx]

| **Species** | | **Events per species per day** | | | | | | |
| --- | --- | --- | --- | --- | --- | --- | --- | --- |
| **Scientific name** | **Common name** | **Ruipa** | **Idete** | **Mbatwa** | **Lumemo** | | **Ndundulu-Luhomero** | **Mwanihana** |
| *Cephalophus harveyi* | Harvey's duiker | 259 | 210 | 117 | 68 | 73 | | 175 |
| *Cephalophus spadix* | Abbott's duiker | 2 | 7 | 0 | 0 | 23 | | 17 |
| *Cerocebus sanjei* | Sanje mangabey | 0 | 0 | 0 | 0 | 0 | | 43 |
| *Cricetomys ansorgei* | Giant pouched rat | 15 | 12 | 0 | 22 | 28 | | 47 |
| *Hippotragus niger* | Sable antelope | 0 | 0 | 1 | 0 | 0 | | 0 |
| *Kobus ellipsiprymnus* | Waterbuck | 3 | 0 | 0 | 5 | 0 | | 0 |
| *Madoqua kirkii* | Ugogo dikdik | 0 | 0 | 72 | 0 | 0 | | 0 |
| *Neotragus moschatus* | Suni | 26 | 71 | 6 | 24 | 27 | | 35 |
| *Oreotragus oreotragus* | Klipspringer | 0 | 0 | 7 | 0 | 0 | | 0 |
| *Papio cynocephalus* | Yellow baboon | 8 | 9 | 104 | 26 | 0 | | 6 |
| *Phacochoerus africanus* | Warthog | 0 | 0 | 6 | 2 | 0 | | 0 |
| *Philantomba monticola* | Blue duiker | 1 | 1 | 0 | 0 | 37 | | 0 |
| *Potamochoerus larvatus* | Bush pig | 35 | 54 | 14 | 30 | 37 | | 45 |
| *Sylvicapra grimmia* | Bush duiker | 0 | 0 | 2 | 8 | 0 | | 0 |
| *Syncerus caffer* | Cape buffalo | 4 | 2 | 7 | 23 | 15 | | 13 |
| *Thryonomys swinderianus* | Marsh cane rat | 1 | 0 | 0 | 0 | 4 | | 0 |
| *Tragelaphus scriptus* | Bushbuck | 27 | 4 | 104 | 60 | 28 | | 49 |
| *Tragelaphus strepsiceros* | Greater kudu | 0 | 0 | 37 | 0 | 0 | | 0 |
