# Supplementary material for: Reserve size and anthropogenic disturbance affect the density of an African leopard (Panthera pardus) meta-population
Source: PLoS One. 2019 Jun 12;14(6):e0209541. doi: 10.1371/journal.pone.0209541 (PMC6561539; doi:10.1371/journal.pone.0209541)
Supplement: S3 Table — Posterior Bayesian distributions and quantiles of mean occupancy (ψ) and detectability (p) for the community of potential leopard prey species detected by camera trapping in the Udzungwa mountains of Tanzania (see text for details). (DOCX) [file pone.0209541.s004.docx]

|  | Mean | SD | 2.5% | 97.50% |
| --- | --- | --- | --- | --- |
| Mean *p* | 0.093 | 0.012 | 0.070 | 0.119 |
| Mean *ψ* [Ruipa] | 0.086 | 0.064 | 0.015 | 0.255 |
| Mean *ψ* [Idete] | 0.073 | 0.055 | 0.012 | 0.220 |
| Mean *ψ* [Mbatwa] | 0.109 | 0.073 | 0.025 | 0.301 |
| Mean *ψ* [Lumemo] | 0.102 | 0.071 | 0.018 | 0.287 |
| Mean *ψ* [Ndundulu-Luhomero] | 0.076 | 0.058 | 0.012 | 0.229 |
| Mean *ψ* [Mwanihana] | 0.081 | 0.059 | 0.014 | 0.237 |
