# Supplementary material for: Reserve size and anthropogenic disturbance affect the density of an African leopard (Panthera pardus) meta-population
Source: PLoS One. 2019 Jun 12;14(6):e0209541. doi: 10.1371/journal.pone.0209541 (PMC6561539; doi:10.1371/journal.pone.0209541)
Supplement: S4 Table — List of competing encounter models with the specific hypotheses tested on the baseline encounter probability (p0) and the scale parameter (σ). (DOCX) [file pone.0209541.s005.docx]

| ***p0*** | | | **σ** | | |
| --- | --- | --- | --- | --- | --- |
| Distance to river |  |  | Trap array |  |  |
| Null |  |  | Trap array |  |  |
| Distance to river | + | Distance to boundary | Trap array |  |  |
| Distance to boundary |  |  | Trap array |  |  |
| Camera trap type |  |  | Trap array |  |  |
| Distance to river |  |  | Prey encounter index |  |  |
| Distance to river | + | Distance to boundary | Prey encounter index |  |  |
| Distance to river |  |  | Prey encounter index | + | Distance to river |
| Distance to boundary |  |  | Prey encounter index |  |  |
| Trap array |  |  | Trap array | + | Distance to boundary |
| Distance to boundary |  |  | Prey encounter index | + | Distance to river |
| Trap array |  |  | Trap array |  |  |
| Null |  |  | Null |  |  |
| Distance to river |  |  | Distance to river |  |  |
| Trap array | + | Distance to boundary | Trap array |  |  |
| Trap array | + | Distance to river | Trap array |  |  |
| Distance to river | + | Distance to boundary | Distance to river |  |  |
| Distance to river | + | Distance to boundary | Distance to boundary |  |  |
| Trap array | + | Camera trap type | Trap array |  |  |
| Trap array |  |  | Trap array | + | Distance to river |
| Distance to boundary |  |  | Distance to river |  |  |
| Trap array |  |  | Prey encounter index |  |  |
| Distance to boundary |  |  | Distance to boundary |  |  |
| Trap array |  |  | Null |  |  |
| Trap array |  |  | Distance to river |  |  |
| Trap array |  |  | Distance to boundary |  |  |
| Sex |  |  | Trap array |  |  |
| Distance to river |  |  | Sex | + | Trap array |
| Null |  |  | Sex | + | Trap array |
| Sex |  |  | Sex | + | Trap array |
| Distance to boundary |  |  | Sex | + | Trap array |
| Camera trap type |  |  | Sex | + | Trap array |
| Distance to river | + | Distance to boundary | Prey encounter index | + | Sex |
| Trap array |  |  | Trap array | + | Sex |
| Distance to river |  |  | Sex |  |  |
| Distance to river | + | Distance to boundary | Sex |  |  |
| Null |  |  | Sex |  |  |
| Distance to river | + | Distance to boundary | Distance to river | + | Sex |
| Distance to boundary |  |  | Sex |  |  |
| Distance to river | + | Distance to boundary | Distance to boundary | + | Sex |
| Camera trap type |  |  | Sex |  |  |
| Trap array | + | Distance to river | Sex |  |  |
| Trap array |  |  | Sex |  |  |
| Trap array | + | Camera trap type | Sex |  |  |
