# Supplementary material for: Reserve size and anthropogenic disturbance affect the density of an African leopard (Panthera pardus) meta-population
Source: PLoS One. 2019 Jun 12;14(6):e0209541. doi: 10.1371/journal.pone.0209541 (PMC6561539; doi:10.1371/journal.pone.0209541)
Supplement: S5 Table — List of competing models for leopard density, as the combination of different covariates in the density linear predictor. (DOCX) [file pone.0209541.s006.docx]

| **Density** | | | ***p*0** | **σ** |
| --- | --- | --- | --- | --- |
| Distance to boundary |  |  | Distance to river | Trap array |
| Prey encounter index | + | Distance to boundary | Distance to river | Trap array |
| Null |  |  | Distance to river | Trap array |
| Elevation |  |  | Distance to river | Trap array |
| Prey encounter index |  |  | Distance to river | Trap array |
| Distance to river |  |  | Distance to river | Trap array |
| Prey encounter index | + | Elevation | Distance to river | Trap array |
| Prey encounter index | + | Distance to river | Distance to river | Trap array |
| Trap array | + | Distance to boundary | Distance to river | Trap array |
| Trap array |  |  | Distance to river | Trap array |
| Trap array | + | Distance to river | Distance to river | Trap array |
| Trap array | + | Elevation | Distance to river | Trap array |
| Sex |  |  | Distance to river | Trap array |
