# Supplementary material for: Reserve size and anthropogenic disturbance affect the density of an African leopard (Panthera pardus) meta-population
Source: PLoS One. 2019 Jun 12;14(6):e0209541. doi: 10.1371/journal.pone.0209541 (PMC6561539; doi:10.1371/journal.pone.0209541)
Supplement: S6 Table — Estimates are expressed in meters (m) with upper and lower confidence intervals (CI). (DOCX) [file pone.0209541.s007.docx]

| **Trap array** | **Mean (m)** | **SE (m)** | **Lower 95% CI (m)** | **Upper 95% CI (m)** |
| --- | --- | --- | --- | --- |
| Idete | 1840 | 266 | 1495 | 2264 |
| Lumemo | 2440 | 307 | 2038 | 2922 |
| Mbatwa | 1901 | 194 | 1642 | 2201 |
| Mwanihana | 1149 | 171 | 928 | 1422 |
| Ndundulu | 1298 | 142 | 1109 | 1520 |
| Ruipa | 2261 | 271 | 1905 | 2685 |
