# Supplementary material for: Reserve size and anthropogenic disturbance affect the density of an African leopard (Panthera pardus) meta-population
Source: PLoS One. 2019 Jun 12;14(6):e0209541. doi: 10.1371/journal.pone.0209541 (PMC6561539; doi:10.1371/journal.pone.0209541)
Supplement: S7 Table — 95% home-range estimates in km2 for leopards in the Udzungwa mountains of Tanzania in each trap array based on spatially explicit capture-recapture models. (DOCX) [file pone.0209541.s008.docx]

| **Study site** | **km^2^** | **Quartiles and mean** | |
| --- | --- | --- | --- |
| Mwanihana | 24.83 | Minimum | 24.84 |
| Ndundulu-Luhomero | 31.72 | 1st quartile | 39.71 |
| Idete | 63.68 | Median | 65.84 |
| Mbatwa | 68 | Mean | 66.09 |
| Ruipa | 96.23 | 3rd quartile | 89.18 |
| Lumemo | 112.07 | Maximum | 112.07 |
